# The Phylogenetic Origin of *oskar* Coincided with the Origin of Maternally Provisioned Germ Plasm and Pole Cells at the Base of the Holometabola

**DOI:** 10.1371/journal.pgen.1002029

**Published:** 2011-04-28

**Authors:** Jeremy A. Lynch, Orhan Özüak, Abderrahman Khila, Ehab Abouheif, Claude Desplan, Siegfried Roth

**Affiliations:** 1 Institute for Developmental Biology, University of Cologne, Cologne, Germany; 2 Department of Biology, McGill University, Montreal, Canada; 3 Center for Developmental Genetics, Department of Biology, New York University, New York, New York, United States of America; University of California Davis, United States of America

## Abstract

The establishment of the germline is a critical, yet surprisingly evolutionarily labile, event in the development of sexually reproducing animals. In the fly *Drosophila*, germ cells acquire their fate early during development through the inheritance of the germ plasm, a specialized maternal cytoplasm localized at the posterior pole of the oocyte. The gene *oskar* (*osk*) is both necessary and sufficient for assembling this substance. Both maternal germ plasm and *oskar* are evolutionary novelties within the insects, as the germline is specified by zygotic induction in basally branching insects, and *osk* has until now only been detected in dipterans. In order to understand the origin of these evolutionary novelties, we used comparative genomics, parental RNAi, and gene expression analyses in multiple insect species. We have found that the origin of *osk* and its role in specifying the germline coincided with the innovation of maternal germ plasm and pole cells at the base of the holometabolous insects and that losses of *osk* are correlated with changes in germline determination strategies within the Holometabola. Our results indicate that the invention of the novel gene *osk* was a key innovation that allowed the transition from the ancestral late zygotic mode of germline induction to a maternally controlled establishment of the germline found in many holometabolous insect species. We propose that the ancestral role of *osk* was to connect an upstream network ancestrally involved in mRNA localization and translational control to a downstream regulatory network ancestrally involved in executing the germ cell program.

## Author Summary

The establishment of the germline during embryogenesis is a critical milestone for sexually reproducing organisms, but one that is surprisingly labile in evolution. For example, in the fly *Drosophila*, the germline is set aside early in embryogenesis due to the localized synthesis of the germ plasm at the posterior pole of the oocyte, and the gene *oskar* is both necessary and sufficient for assembly of the germ plasm. However, *oskar* orthologs have not been found outside of flies and mosquitos, while the maternal provisioning of germ plasm and the early setting aside of the germline are unique to, but not universal within, the holometabolous insects. In order to understand how the novel mode of germline determination found in *Drosophila* could have evolved, we have examined this process in the wasp *Nasonia*. Our results indicate that the phylogenetic origin of the insect mode of maternal germ plasm provision and early establishment of the germline coincided with the origin *oskar* at the base of the holometabolous insects. Our results further suggest that *osk* was independently lost in multiple holometabolous insect lineages and that these losses are phylogenetically correlated with changes in germline determination strategies in these species.

## Introduction

Germ cells are essential for the transfer of heritable information and, therefore, the determination of their fate is a critical event in the development and evolution of sexually reproducing organisms. Two general strategies for generating the germline have evolved in animals: cytoplasmic inheritance or zygotic induction. Inheritance requires that determinants of the germ cell fate (mRNAs and proteins that form the pole plasm) are maternally generated and provisioned to the oocyte. In contrast, induction involves the acquisition *de novo* of the germ cell fate in a subset of cells later during embryonic development [1], [2].

Some of the first experiments that proved the existence of a maternally generated substance capable of inducing the germline fate were conducted in insects. It had been observed that in many insect species, a distinct region of cytoplasm (called pole plasm, or oosome) is localized to the posterior pole of the oocyte during oogenesis. This pole plasm remains at the posterior during early embryogenesis, until cleavage nuclei reach the embryo cortex. Those nuclei that reach the posterior pole of the embryo interact with the pole plasm, bud from the posterior pole, and become cellularized precociously in comparison to the other blastodermal nuclei [3]. These cells are termed pole cells, and will give rise to the germline [4], [5]. Classical embryonic manipulations showed that the pole plasm is both necessary [6], and sufficient [7] to produce the primordial germ cells.

Genetic analyses have identified numerous molecular factors that are required for the proper production of the pole plasm and pole cells in *Drosophila*. Only one of these, *oskar* (*osk*), is both necessary and sufficient to induce the production of polar granules and pole cells [8]. Due to the sufficiency of Osk to induce germ plasm, it must be tightly regulated to prevent ectopic induction of germline fate. To this end, genes upstream of *osk* are generally required to regulate translation of *osk* mRNA and to mediate its transport between the time it is transcribed in the nurse cells and the time it is properly posteriorly localized in the oocyte [9]. Genes downstream of *osk* are generally required to assemble the polar granules or to mediate proper behavior of the pole cells [9], and have highly conserved functions in the germline throughout the Metazoa [10]–[12].

**Figure 1:**
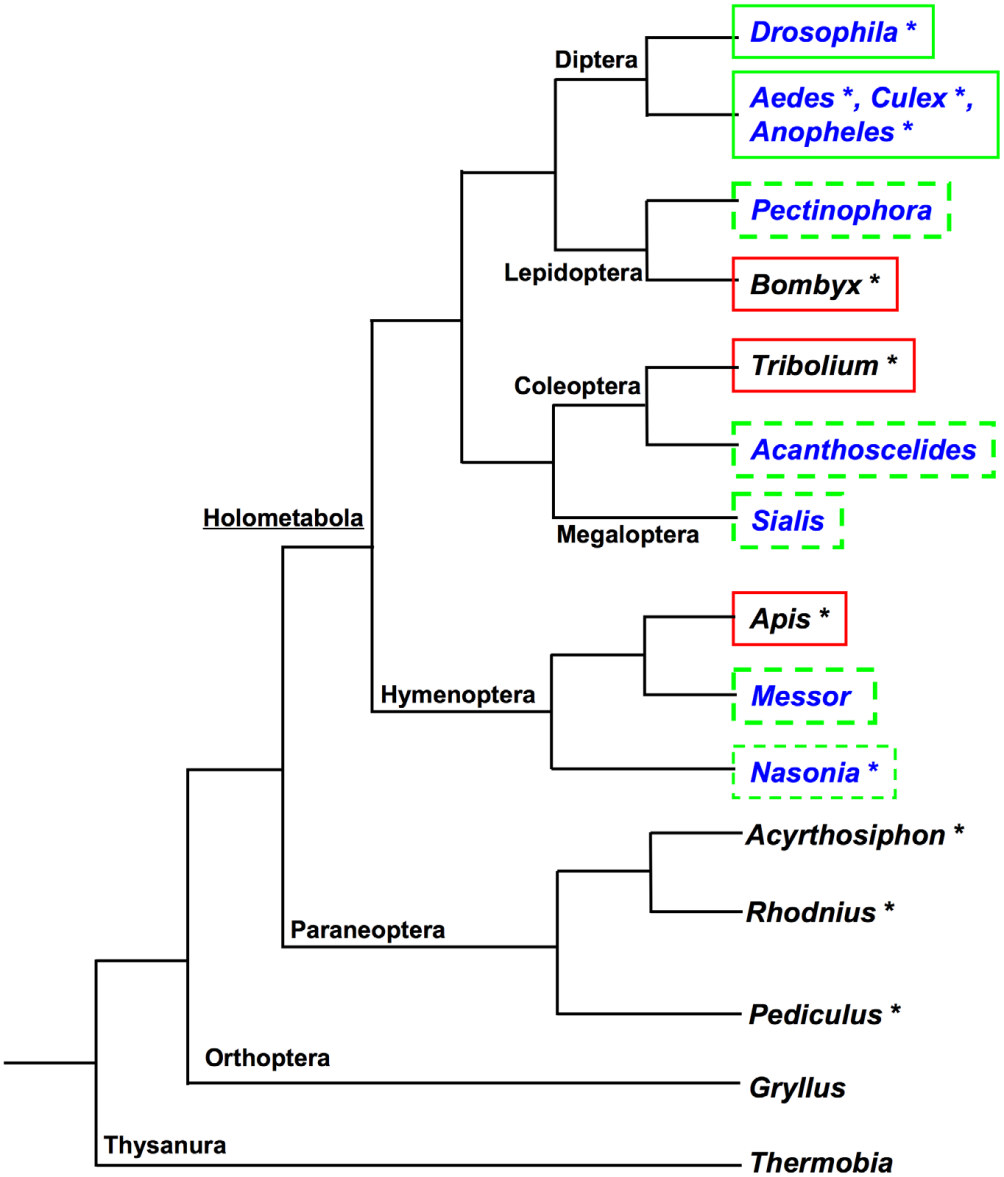
Current understanding of the distribution of maternal germ plasm, pole cells, and *oskar* orthologs in the insects. Genus names in blue are those in which maternal germ plasm and pole cells have been described. Asterisks indicate a sequenced genome. Green boxes indicate confirmed presence of *osk*. Red boxes indicate apparent absence of *osk* in the genome. Dashed green box indicates the hypothesis that species with posteriorly localized maternal germ plasm and pole cells require a factor with Osk-like function and regulation.

Current data suggest that the mode of germline determination found in *Drosophila* is not the ancestral mode among the insects. So far neither unequivocal maternal germ plasm nor pole cells have been detected in representatives of basally branching hemimetabolous insect orders. Rather, species from these orders instead appear to rely on zygotic induction mechanisms to specify their germline [13]–[17] (Figure 1). Consistent with absence of cytoplasmic inheritance of germline determinants and the production of pole cells, the processes for which *osk* is required, orthologs of *osk* have not been detected in any of the sequenced genomes of the hemimetabolous insects *Acyrthosiphon pisum* [18], *Rhodnius prolixis* (http://genome.wustl.edu/genomes/view/rhodnius_prolixus/), and *Pediculus humanus* http://phumanus.vectorbase.org/SequenceData/Genome/ (Figure 1, Table S1).

Among the Holometabola, *osk* orthologs are also apparently absent from the sequenced genomes of the silk moth *Bombyx mori* (Lepidoptera) [19], the beetle *Tribolium castaneum* (Coleoptera) [20], and the honeybee *Apis mellifera* (Hymenoptera) [21] (Figure 1, Table S1). Consistent with this absence *osk*, *Bombyx*, *Tribolium*, and *Apis* all also lack maternal germ plasm, do not produce pole cells, and appear to rather use zygotic inductive strategies to generate the germline [22]–[25] (Figure 1).

These observations led to the idea that *osk* may have been a novelty that originated within the dipteran lineage [26], [27]. However, *Drosophila*-like modes of germline determination through posteriorly localized maternal germ plasm and pole cells are also found throughout the Holometabola, including most major lineages of the Hymenoptera (e.g., *Nasonia vitripennis* [28] sawflies [29] and multiple ant species [30], [31]), the Coleoptera (e.g., *Acanthoscelides obtectus* [32], *Dermestes frischi* [33]), Megaloptera (*Sialis misuhashii* [34]) and Lepidoptera (*Pectinophora gossypiella* [35]) (Figure 1). Despite the similarity of the strategies for germline determination in the above species to that employed in *Drosophila*, *osk* orthologs have only been identified in the genomes of the dipterans *Anopheles gambiae*, *Aedes aegypti*, and *Culex pipiens* [36], [37] (Figure 1).

These observations raised the question of evolutionary origin of *osk* in the insects and whether or not this gene is associated with the evolution of the inheritance mode of germline specification. To answer these fundamental questions, we examined the molecular basis of maternal germ plasm production in the wasp *Nasonia vitripennis*. We chose *Nasonia* because its genome was recently sequenced [38], it is amenable to functional manipulation by pRNAi [39], and its key phylogenetic position within the most basally branching holometabolous order, the Hymenoptera [40], [41]. We show that the regulatory network underlying the production of maternal germ plasm and pole cells is largely conserved between *Nasonia* and *Drosophila*, and argue that these features had a common phylogenetic origin at the base of the Holometabola. In addition, we provide evidence that the possession of an *oskar* ortholog is a general feature of insects that produce pole cells, and that *oskar* has likely been lost independently multiple times within the Holometabola in correlation with shifts in strategies for establishing the germline.

## Results

### Cloning and sequence analysis of Nv-Osk

Attempts to detect a *Nasonia* ortholog by BLAST [42] searches using the *Drosophila* Osk sequence as the query failed to return significant hits. However, using Oskar sequences identified in the mosquitoes *Culex* and *Aedes*, we identified a *Nasonia* genomic region that showed significant similarity to the mosquito sequences. Using the predicted peptide sequence in this region, reciprocal BLAST against the mosquito and *Drosophila* genome databases returned results with significant E-values that corresponded to *osk* genes in each of these species (Table S1). We thus hypothesized that the region in the wasp genome detected by mosquito Osk BLASTs corresponded to *Nasonia osk*, and cloned a 1500 base pair fragment representing the full length complementary DNA of *Nasonia osk* using RACE PCR. This sequence contains an open reading frame that is predicted to generate a protein of 375 amino acids.

**Figure 2:**
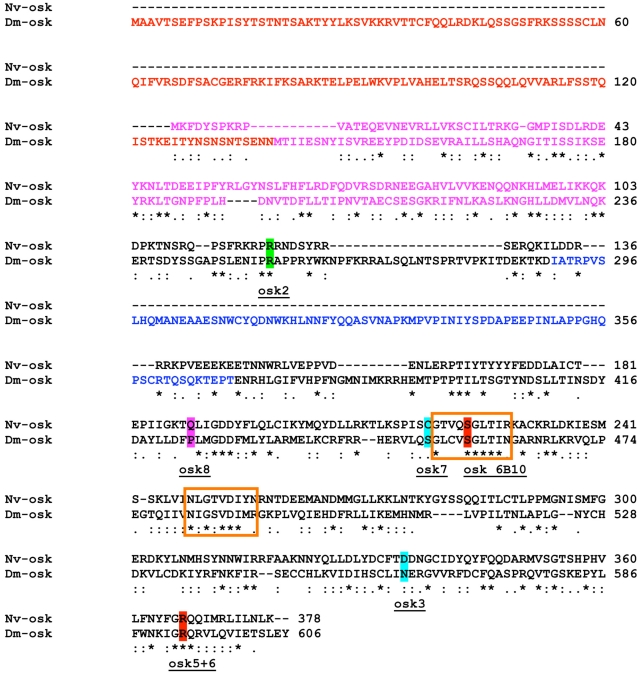
Sequence features of Nv-Osk protein. CLUSTALW generated alignment of *D. melanogaster* and *N. vitripennis* Osk proteins. Red text is the fly long-Osk specific region. Blue indicates the putative LASP binding domain of fly Osk. Pink text indicates the Lotus/Tejas homology domain. The characterized missense mutations in fl*y osk* were mapped on the alignment, and were categorized as follows: green shaded residues are those that are conserved between wasp and fly Osk, but are not conserved in the mosquito sequences (osk2). Red shaded residues are conserved in wasp, mosquito, and fly (osk6B10 and osk 5+6). Pink shading indicates residues that are conserved between wasp and mosquito Osk, but not in *Drosophila* (osk8). Finally, light blue shaded residues are conserved between mosquitoes and fly, but not in the wasp (osk3 and osk7). Orange boxes delineate the putative hydrolase homology domains in *Drosophila* and *Nasonia* Osk.

The overall Nv-Osk sequence is similar to that of *Drosophila* Osk (16% identity, 33% similarity, 44% gaps), and many of the residues critical for fly Osk function are conserved in the *Nasonia* sequence (Figure 2). However, we could identify two regions that appear to be unique to the fly sequence. One is the region that is specific to the *Drosophila* long-Osk isoform [43] (Figure 2, red text). No similarity to this region appears to be encoded in the *Nv-osk* mRNA, nor is it present in mosquito Osk sequences. The other region that is absent in Nv-Osk includes amino acids 290 to 396 in Dm-Osk (Figure 2, blue text), which corresponds to the domain interacting with LASP to regulate Osk anchoring to the actin cytoskeleton [44]. Interestingly, this region is also absent from the mosquito Osk sequences, which appear to be more similar to Nv-Osk in sequence and general structure (*Culex/Nasonia*: 24% identity, 42% similarity, 22% gaps).

A search in the *Conserved Domain Database* indicates that the central portion of the Nv-Osk protein shares similarity with a GDSL/SGNH-hydrolase or lipase-like domain (Figure 2, orange boxes), consistent with similar observations made for *C. pipiens* and *A. aegypti* Osk orthologs [36]. This domain is weakly detected in *Drosophila* Osk and it is not clear whether it is necessary for Osk function in pole plasm assembly.

In addition, the N-terminal region of Nv-Osk shows strong similarity to a domain also present at the N-termini of highly conserved tudor-domain containing proteins. This domain has been independently identified *in silico* as either the Lotus domain [45], or Tejas domain [46]. This domain is present at the N-terminus of orthologs of *tudor-domain-containing-7* and *-5* (*tdrd7*, *tdrd5*), and related tudor domain containing genes [47], and is detected only weakly in fly Osk. *tdrd7* and *tdrd5* orthologs are found throughout the Metazoa, including all sequenced insect genomes (JAL, personal observation), and are characterized by the presence of Tudor domains toward the C-terminus of the protein, which are absent in Osk proteins. The N-terminal 100 amino acids of Nv-Osk show strong homology to Tdrd7 orthologs throughout the Metazoa, ranging from 39% identical (BLAST E-value 8e-09) to the *Apis* ortholog, 31% identical (BLAST E-value 1e-05) to the *Hydra* ortholog, and 29% identical (BLAST E-value 7e-05) for the *Danio* (zebrafish) ortholog. In comparison, the *Apis* and *Danio* Tdrd7 C-termini are 49% identical (BLAST E-value 2e-13), and *Apis* and *Hydra* proteins are 30% identical (BLAST E-value 3e-09) in the N-terminal region.

In zebrafish, *tdrd7* has a role in controlling germ granule morphology and number during embryogenesis [48]. Furthermore, the *Drosophila tdrd5* ortholog, *tejas*, has a critical role in germline development, and the N-terminal region of this protein (including the Tejas domain, which is similar to the N-terminus of Nv-Osk) has been shown to physically interact with Vas [46]. Finally, a bioinformatic analysis of proteins containing domains similar to those found in Osk and Tdrd7/5 N-termini (termed by the authors OST-HTH) indicated that these domains may bind double-stranded RNA [49]. These results indicate that Oskar is at least partially related to genes that had ancestral germline and/or RNA binding functions.

### *Nv-osk* is expressed in the germline and is localized to the posterior of the oocyte and early embryos

**Figure 3:**
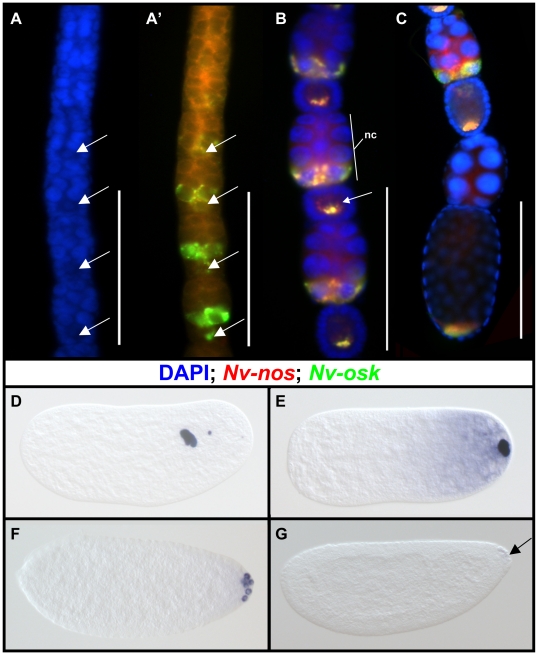
Expression of *Nv-osk* during oogenesis and embryogenesis. During oogenesis (A–C) and embryogenesis (D–G). A, A′: Expression of *Nv-osk* (green) and *Nv-nos* (red) in early oogenesis. Arrows mark oocyte. B: Later stage of oogenesis, after completion of encapsulation of the oocyte by follicle cells. nc = nurse cells. C: Toward the end of oogenesis, most *Nv-osk* mRNA is rapidly dumped from the nurse cells into the oocyte (compare lower egg chamber to the upper). D: Embryo in division cycle 2–3 stained for *Nv-osk*. E: Embryo just before syncytial blasotoderm formation. F: Embryo in early syncytial blastoderm stage. G: Embryo just before cellularization of the blastoderm. Scale bars = 100 micrometers.

*Nasonia* oogenesis occurs in ovarioles of the polytrophic-meroistic type, where each oocyte is associated with its own population of nurse cells, and has been described in detail previously [50]. *Nv-osk* mRNA is detected quite early in oogenesis, just after the time that the nurse cells become distinguishable from the oocyte (Figure 3A, 3A′). As the egg chambers mature (Figure 3B), *Nv-osk* is expressed at very high levels in only the posterior nurse cells nearest to the oocyte. Within these cells, *Nv-osk* mRNA is incorporated into particles (Figure 3B), a pattern similar to that of *Nv-otd1* [51]. From the very early stages of oogenesis, *Nv-osk* is transported from the nurse cells to the oocyte, where it is localized to the posterior pole in a pattern similar to that of *Nv-nos* (Figure 3A′, 3B, 3C). During late oogenesis, *Nv-osk* mRNA levels go from high to barely detectable in the nurse cells of adjacent egg chambers (Figure 3C). This likely indicates the onset of nurse cell dumping, as from this point on the nurse cells will become progressively smaller and eventually disappear. This pattern of rapid transfer of mRNA is similar to what is seen for *Nv-otd1* during late oogenesis, except that *Nv-otd1* mRNA accumulates at the anterior pole of the oocyte at this stage [51].

In the early embryo, *Nv-osk* mRNA remains localized to the posterior pole, and most of the mRNA is associated with the oosome, a large, discreet structure associated with the posterior pole. The oosome migrates within the embryo during the early cleavages (Figure 3D), before returning to the posterior pole just before the formation of pole cells (Figure 3E, see [51] for details). At this stage, a population of *Nv-osk* mRNA not contained within the oosome is observed in a gradient at the posterior pole, a pattern which is typical for oosome associated mRNAs (e.g., *otd1*and *nanos* in *Nasonia* [52]). *Nv-osk* mRNA still associated with the oosome is then incorporated into the pole cells (Figure 3F), while the cytoplasmic population remains in the embryo proper (not shown, but see [51] for expression of *Nv-nos* mRNA, which shows identical behavior at these stages). Both populations of mRNA are finally degraded as the cellular blastoderm begins to form (Figure 3G).

### *Nv-osk* is required for oosome assembly and pole cell formation

We used parental RNA interference (pRNAi) to analyze the function of *Nv-osk* during *Nasonia* development. We obtained specific phenotypes that vary in terms of intensity allowing us to infer a number of potential functions for *Nv-osk* during oogenesis and early embryogenesis.

**Figure 4:**
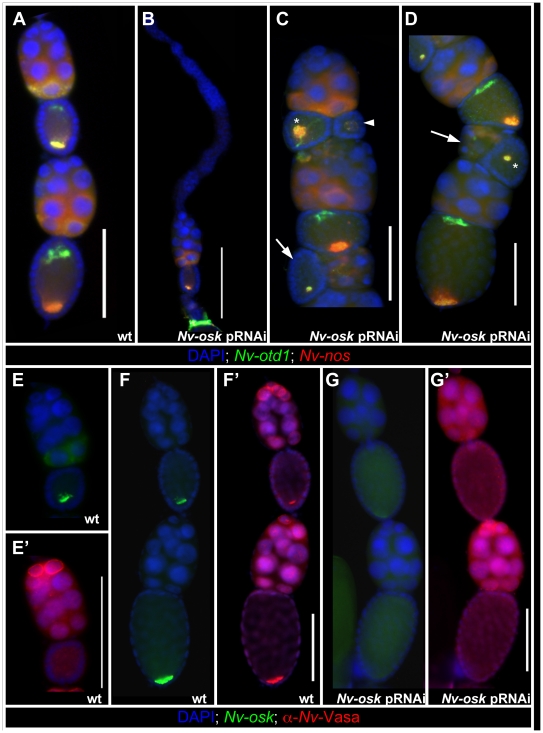
Effects of *Nv-osk* pRNAi during oogenesis. A: Wild type *Nasonia* ovariole stained with *Nv-otd1* (green) *Nv-nos* (red), and DAPI (blue). B: Strong *Nv-osk* pRNAi knockdown , very few mature egg chambers are formed. C,D: In weaker *Nv-osk* pRNAi knockdowns the linear arrangement of egg-chambers is severely disrupted. Egg chambers in reverse orientation (arrowhead) or perpendicular to the AP axis of the ovariole (arrows) are observed. Within the oocytes, axial polarity (asterisks) and mRNA localization (arrowhead in C) defects occur. E, E′: In wild type, Nv-Vas protein is not localized in young oocytes (E′) even though high levels of *Nv-osk* mRNA are localized at the posterior pole (E). Nv-Vas protein appears to be concentrated on the surface of the most anterior nurse cell nuclei. F, F′: *Nv-osk* mRNA (F) and Nv-Vas (F′) accumulation late in oogenesis. G, G′: Expression of *Nv-osk* (G) and Nv-Vas (G′) after *Nv-osk* pRNAi.

In ovarioles showing the strongest *Nv-osk* pRNAi effect, only a few egg chambers are produced (Figure 4B, compare to 4A) indicating that *Nv-osk* has an early role in promoting oogenesis. This may be related to a similar phenotype produced by mRNA null mutations in fly *osk* [53].

The *Nasonia* ovariole normally consists of a linear array of egg chambers, with the oocytes always lying directly posterior to their sister nurse cells and directly anterior to the next older egg chamber (Figure 4A). In the milder phenotypes of *Nv-osk* pRNAi, this linear arrangement is disrupted, and egg chambers arranged perpendicularly to the long axis of the ovariole (arrows in Figure 4C and 4D), or with reversed polarity (arrowhead Figure 4C) are observed. Egg chamber polarity defects are also observed after pRNAi against *Nv-vas* (not shown) and *Nv-tud* (see below), indicating that there is a novel role for germ plasm components in establishing polarity of egg chambers within the ovarioles of *Nasonia*. Due to the variability in the final morphology of ovarioles after pRNAi for *Nv-vas*, *-osk*, and *-tud*, it is not clear whether these phenotypes are all the result of the disruption of a single developmental process.

Within the oocytes, *Nv-nos* and *otd1* mRNAs are sometimes localized more loosely than normal (asterisk and arrowhead Figure 4C) or mislocalized in relation to the AP axis of the oocyte (asterisk Figure 4D) after *Nv-osk* pRNAi. These phenotypes may represent a disruption of the internal polarity of the oocytes and/or proper anchoring of localized mRNAs. A more detailed understanding of oocyte cytoskeletal polarity and mRNA anchoring mechanisms in *Nasonia* will be required to resolve this uncertainty. In any case, these results indicate that *Nv-osk* is required for germline development, for establishing the polarity of the egg chambers, and for the proper localization of the pole plasm to the posterior pole.

In *Drosophila*, the recruitment of Vas protein to the posterior pole of the oocyte by Osk is a critical step in polar granule assembly. To test whether Nv-Osk functions in a similar way, we examined the distribution of Nv-Vas using a *Nasonia* specific Vasa antiserum in wild type and *Nv-osk* pRNAi ovaries. During early oogenesis, Nv-Vas protein is detected primarily on the surface of the nuclei of the most anterior nurse cells (Figure 4E′). This is consistent with the strong transcription of *Nv-vas* detected in these cells (Figure S1A). Localized Nv-Vas protein is not seen in early oocytes (Figure 4E′), even though *Nv-osk* is already localized at high levels at the posterior (Figure 4E). Localized Nv-Vas becomes visible in the oocyte relatively late in oogenesis, when the oocyte is of the same size as the nurse cell cluster (Figure 4F, 4F′). This accumulation of Nv-Vas at the posterior pole is abolished after *Nv-osk* pRNAi (Figure 4G), while Nv-Vas production in anterior nurse cells appears unaffected (Figure 4G′). Thus, the role of Osk in recruiting germ plasm components to the posterior pole is conserved between *Drosophila* and *Nasonia*.

**Figure 5:**
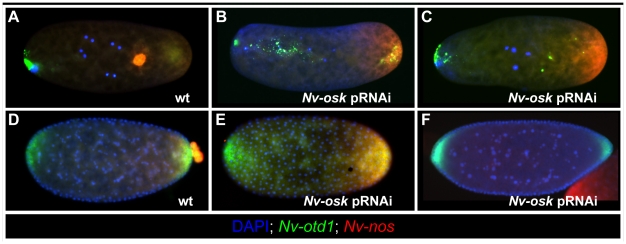
Effects of *Nv-osk* pRNAi during embryogenesis. A: Wild type localization of *Nv-nos* (red) and *Nv-otd1* (green) mRNA in early embryogenesis. B, C: Expression of *Nv-nos* and *Nv-otd1* in early embryos after *Nv-osk* pRNAi. D: Wild type expression of *Nv-nos* and *Nv-otd1* just after pole cell formation. E: Expression of *Nv-nos* and *Nv-otd1* in an *Nv-osk* pRNAi embryo at a stage similar to D. F: Expression of *Nv-nos* and *Nv-otd1* in *Nv-osk* pRNAi embryo just before cellularization.

Posteriorly localized mRNAs (e.g., *Nv-nos*, *Nv-otd1* and *Nv-osk*) are incorporated into the oosome in early *Nasonia* embryos (Figure 5A). After *Nv-osk* pRNAi, these mRNAs remain in a homogenous cap at the posterior pole of the embryo, and the oosome is not formed (100% penetrance, N = 60) (Figure 5B, 5C). In addition, the anterior localization of *Nv-otd1* mRNA is disrupted. Rather than being tightly localized at the anterior pole, *Nv-otd1* mRNA is often seen in particles distributed throughout the anterior half of the embryo (Figure 5B). This part of the phenotype may be related to the polarity defects observed in *Nv-osk* pRNAi oocytes.

pRNAi against *Nv-osk* also results in the completely penetrant (N = 57) loss of pole cells (Compare wild type in Figure 5D to 5E). In the absence of the protective environment of the pole cells, all *Nv-nos* mRNA is lost from the embryo by the late blastoderm stage (Figure 5F). A similar phenomenon is seen after *Nv-vas* pRNAi [51]. *Nv-osk* pRNAi also causes embryonic patterning phenotypes that result in larval lethality (42%, N = 75). Only a portion (13%) showed phenotypes similar to *Nv-nos* pRNAi [51], while the remainder of affected cuticles showed defects in head patterning, or more severe patterning disruptions of unclear origin. This range of phenotype was also seen for *Nv-vasa* [51], and these observations indicate that the roles of *Nasonia* germ plasm assembly factors in embryonic patterning are much more complicated than they are in the fly, where *nos* mRNA translation is the main embryonic patterning output of germ plasm assembly [54].

### *Nv-osk* function is upstream of *Nv-vas* and *Nv-tud*

**Figure 6:**
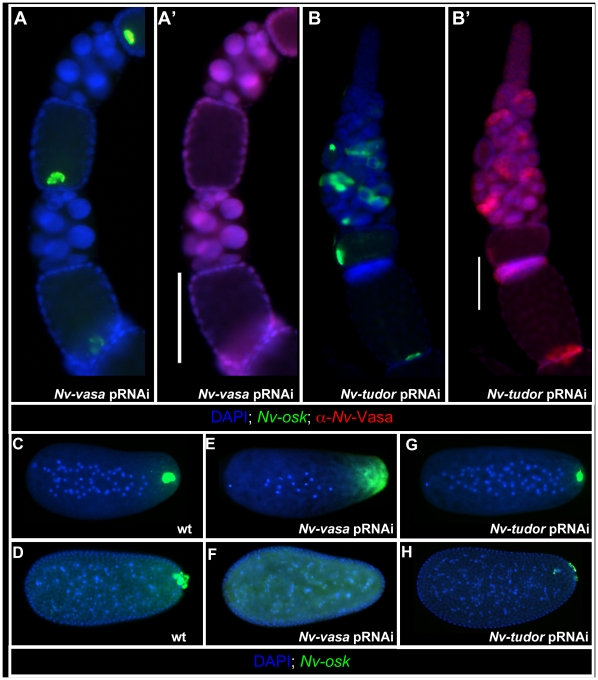
Function of *Nv-vas* and *Nv-tud* in oosome formation and *Nv-osk* localization. A, A′: After *Nv-vas* pRNAi, late oocytes show a looser localization of *Nv-osk* mRNA at the posterior pole, and no accumulation of Nv-Vas is seen in the oocyte (compare to wild type in Figure 4F, 4F′). B, B′: After *Nv-tud* pRNAi, the polarity of the egg chambers within the ovariole can often be disturbed. In spite of this, Nv-Vas still accumulates at the posterior pole, and *Nv-osk* mRNA localization appears normal. C: Wild type expression of *Nv-osk* during early syncytial divisions. D: Wild type *Nv-osk* expression just after pole cell formation. E: *Nv-osk* expression in early *Nv-vas* pRNAi embryo. F: *Nv-vas* pRNAi embryo at stage similar to D. G: Early cleavage stage *Nv-tud* pRNAi embryo. H: *Nv-tud* pRNAi early blastoderm embryo.

In *Drosophila*, Oskar acts through two main downstream proteins to produce polar granules: Vas and Tud [9]. As shown above, Nv-Osk functions upstream of Nv-Vas recruitment to the posterior during oogenesis (Figure 4G′). However, the functional relationship between Nv-Osk and Nv-Vas in the ovary may not be strictly hierarchical, as Nv-Vas knockdown (Figure 6A′) leads to defects in the proper anchoring and tight localization of *Nv-osk* mRNA to the posterior pole of the oocyte (Figure 6A). In the embryo, *Nv-vasa* pRNAi results in the completely penetrant loss of the oosome (Figure 6E) and pole cells (Figure 6F), similar to the effects of *Nv-osk* pRNAi.

In contrast to *Nv-osk* and *Nv-vas* pRNAi, knockdown of *Nv-tud*, which is expressed weakly and ubiquitously in the nurse cells and oocyte (Figure S1B), has only a minor effect on posterior accumulation of Nv-Vas protein in the oocyte, even when strong polarity defects within the ovariole are observed (Figure 6B, 6B′). In the embryo, the oosome is still formed, but is significantly reduced in size (Compare Figure 6G to 6C). In line with these apparently weaker effects, *Nv-tud* pRNAi leads to a reduction in the number of pole cells, and those that do form are smaller, less spherical, and less segregated from the somatic nuclei at the posterior pole which may indicate that they are not completely differentiated as primordial germ cells (Compare Figure 6H to 6D). These results indicate that, similar to fly *tud* [8], [55], *Nv-tud* function is downstream of *Nv-vas* and *Nv-osk* in the production of the germ plasm. However, due to the incompleteness and variability of pRNAi efficiency, we cannot exclude the possibility that the weaker defects are the result of general weaker knockdown of *Nv-tud* with pRNAi.

### Regulation of *Nv-osk* function

In *Drosophila*, the localization and regulation of *osk* translation is tightly regulated in order to prevent ectopic pole plasm and disruptions in segmental patterning. A critical factor in ensuring proper control of *osk* translation is the RNA binding protein Bruno, which binds the UTRs of *osk* mRNA and represses its translation. This repression is relieved under normal circumstances only upon localization of *osk* mRNA to the posterior pole of the oocyte [56]. We analyzed the function of *Nasonia bruno* to test whether a similar mechanism of translational repression operates in *Nasonia* to prevent the ectopic assembly of the oosome.

**Figure 7:**
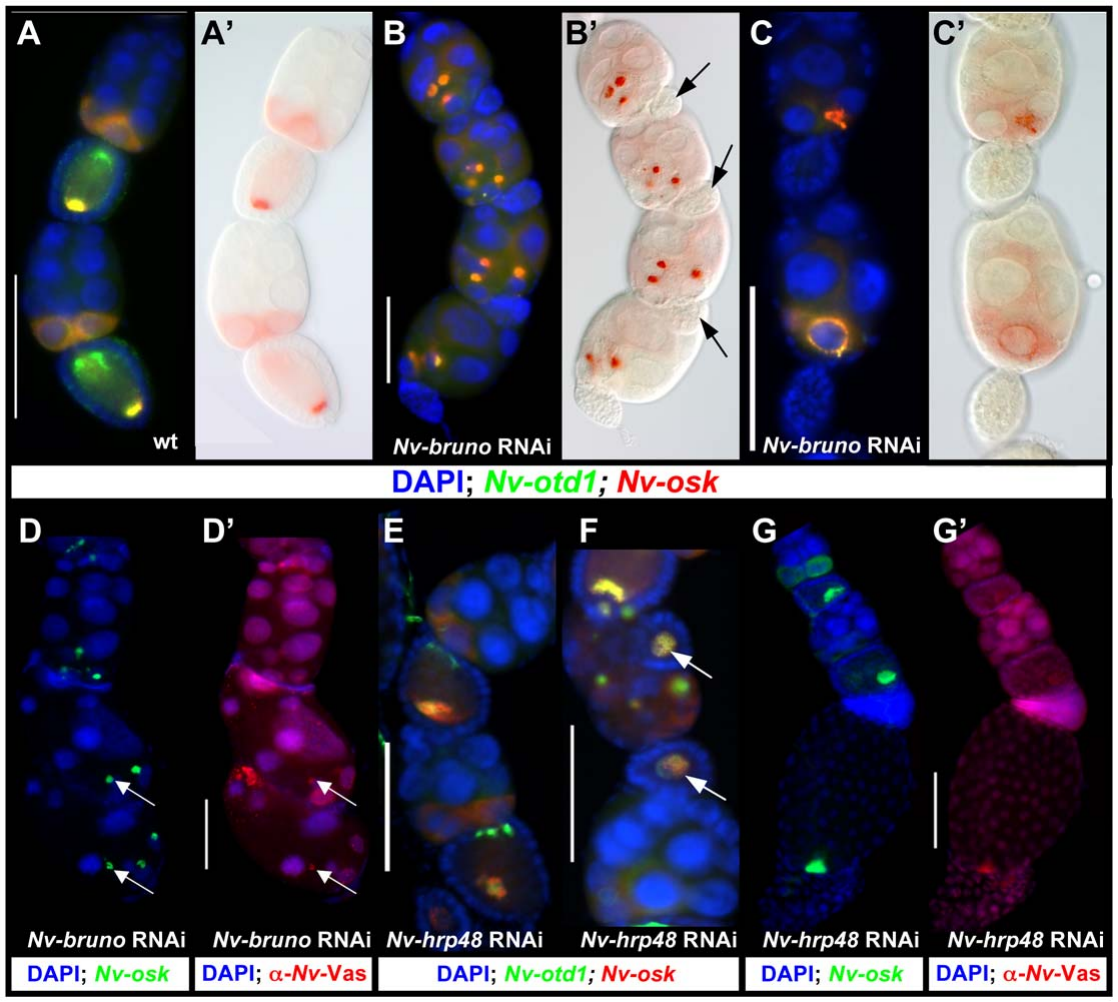
Function of RNA binding proteins in oosome assembly in *Nasonia*. A: Wild type ovarian expression of *Nv-otd1* (green) and *Nv-osk* (red). A′: DIC optical cross section of same egg chambers in A (red = *Nv-osk*). B, B′: Large, dense particles containing *Nv-otd1* and *Nv-osk* mRNA often observed within the nurse cells after *Nv-bruno* pRNAi. C, C′: *Nv-osk* and *Nv-otd1* mRNAs are sometimes concentrated in smaller particles on the surface of the posteriormost nurse cells. D, D′: Ectopic co-localization of *Nv-osk and* Nv-Vas in nurse cells after *Nv-bruno* RNAi. E, F: *Nv-hrp48* pRNAi disrupts the normally tight localization of posteriorly localized mRNAs of *Nv-otd1* and *Nv-osk*. In extreme cases (arrows in F) these mRNAs are completely delocalized. G, G′: *Nv-hrp48* pRNAi only weakly affects Nv-Vas accumulation in *Nasonia* oocytes, despite the looser localization of oosome to the posterior.

In wild-type egg chambers, *Nv-osk* and *otd1* mRNAs are co-expressed in the posterior nurse cells and localized at the posterior pole of the oocyte, while *Nv-otd1* is additionally localized to the anterior pole (Figure 7A, 7A′). The distribution of these mRNAs is dramatically altered after *Nv-bruno* RNAi: both *Nv-osk* and *Nv-otd1* (and *Nv-nos*, data not shown) mRNAs are concentrated in large, dense, spheroid particles in the posterior-most nurse cells (Figure 7B, 7B′). These large particles seem to originate at the nuclear envelope, and smaller particles are observed on the surface of the nurse cell nuclear membranes in some egg-chambers (Figure 7C, 7C′). The morphology (density, large size, spheroidal shape) and molecular composition of the ectopic particles seen after *Nv-bruno* RNAi are similar to the corresponding features of the oosome, indicating that this structure is being ectopically produced in the nurse cells.

If the role of *Nv-bruno* is similar to that of its *Drosophila* ortholog, the production of these oosome-like structures in the nurse cells could be due to the ectopic translation of *Nv-osk* in the nurse cells in the absence of *Nv-bruno*. In support of this conclusion, the large particles are only produced in the most posterior nurse cells nearest to the oocyte, to which *Nv-osk* is restricted (Figure 3), while *Nv-bruno* is expressed in nurse cells located more anteriorly (Figure S1C). However, we cannot exclude that the restriction of large oosome-like particles to the posterior nurse cells is a result of higher levels of Nv-Bruno protein in these cells. In addition, in late *Nv-bruno* pRNAi egg chambers, Nv-Vas protein is associated with the dense accumulation of *Nv-osk* mRNA (Figure 7D), further indicating that oosome formation is being completed ectopically within the nurse cells. Conclusive evidence for a direct role of Nv-Bruno in repressing *Nv-osk* translation will come only with the availability of an antibody against Nv-Osk protein.

Another *Drosophila* RNA binding protein, Hrp48, is critical for both silencing of unlocalized *osk* mRNA translation, and for the proper initiation of its translation once the mRNA is localized to the posterior [57], [58]. *Nv-hrp48* is expressed strongly throughout the nurse cells in the wasp ovary (Figure S1D), and when its function is knocked down, ectopic oosome-like structures are not seen in the nurse cells (Figure 7E, 7F), in contrast to what is seen after *Nv-bruno* pRNAi. In most egg chambers, both *Nv-osk* and *Nv-otd1* mRNAs are expressed normally in the nurse cells, and are transported to the oocyte (Figure 7E). Once in the oocyte, however, these mRNAs do not become localized normally. The extent of mislocalization varies from oocytes that show a looser localization of posterior mRNAs (Figure 7E) to those where *Nv-osk* and *Nv-otd1* mRNAs fail to localize to a distinct cortical location, and are diffusely expressed throughout the smaller than usual oocytes (Figure 7F, arrow). In more weakly affected egg chambers, which have established normal polarity, the pattern of Nv-Vas accumulation appears to be only weakly affected, with the protein appearing at slightly lower levels, and loosely organized, likely reflecting a mild disruption in the proper assembly of the oosome during late oogenesis (Figure 7G, 7G′).

Thus, *Nv-hrp48* appears to have a conserved role in the assembly of the germ plasm in *Nasonia*, and by extension may have a conserved function in regulating the translation of *Nv-osk*. Our results indicate that the primary role of this factor is to promote oosome assembly (and thus, by analogy to *Drosophila*, *Nv-osk* function). However, we cannot completely exclude a second role, such as that seen in *Drosophila*, for *Nv-hrp48* in *Nasonia* in repressing the translation of unlocalized *Nv-osk* in the oocyte [57], [58].

### *osk* is present in a close relative of *Apis*, and likely in a close relative of *Tribolium*

Our results show that a regulatory network of protein interaction centered on Nv-Osk is required for the maternal production of germ plasm, and that this network is highly similar to that found in *Drosophila*. This suggests that, given the basally branching phylogenetic position of the Hymenoptera among the Holometabola, this regulatory network arose in a common ancestor of all Holometabola, and that transitions to the zygotic induction mode of germ cell specification are associated with secondary disruptions of this network. To test this hypothesis, we sought to determine if *osk*, as the central component of this network, is conserved in other species that produce maternal germ plasm and pole cells.

**Figure 8:**
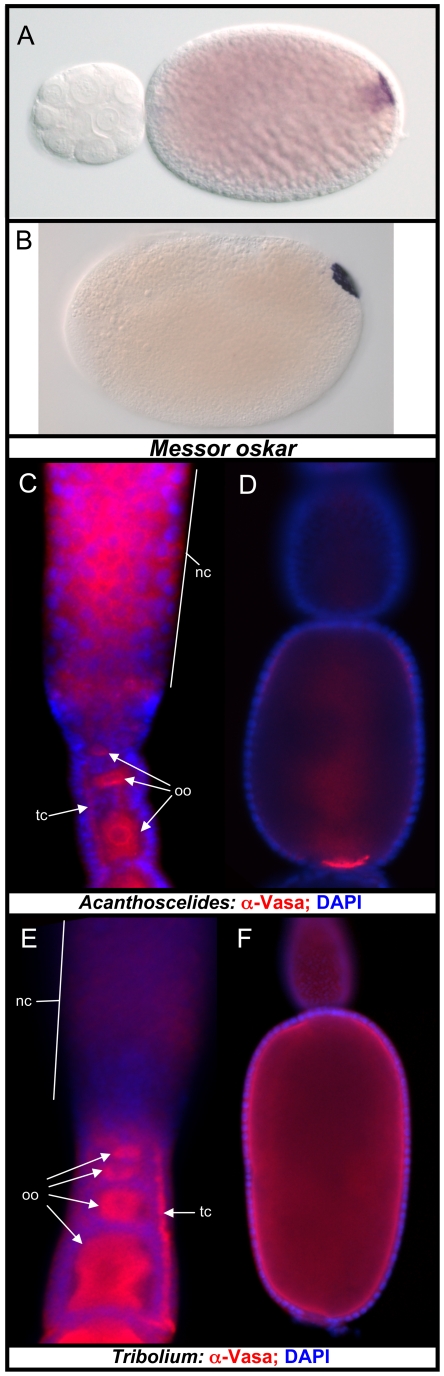
Oskar and oosomes in other Holometabolan species. A, B: An *oskar* ortholog is present in the ant *Messor pergandi*, and is localized posteriorly in an oosome-like structure during oogenesis, and is localized posteriorly during embryogenesis. C: Vas expression in early *Acanthoscelides* oogenesis. nc = nurse cells, tc = trophic cords oo = oocyte. D: Vas localization in a late *Acanthoscelides* oocyte. E: Vas expression in early *Tribolium* oogenesis. F: Vas expression in a late *Tribolium* oocyte.

Multiple ant species have been shown to specify their pole cells through the assembly of a posterior pole plasm that is incorporated into pole cells during early embryogenesis [30], [31]. Consistent with our hypothesis, we successfully cloned an *osk* ortholog in the ant *Messor pergandei*, whose protein sequence shows 46.4% similarity to that of Nv-Osk. Moreover, *Messor osk* (*Mp-osk*) mRNA is localized to the posterior pole of the oocyte during oogenesis (Figure 8A), and embryogenesis (Figure 8B). This pattern of *Mp-osk* mRNA accumulation is similar to that of insects that specify germ cell through cytoplasmic inheritance (e.g., *Nasonia* and *Drosophila*), and suggests that its function in germ cell specification is conserved in ants. In addition, the localization of *Mp-osk* corresponds well to the previously observed localization of Vasa protein and *nanos* mRNA in the oocyte and embryo at equivalent stages in *Messor* and other closely related ant species [30], [31]. *Messor* is a much closer relative of *Apis* than is *Nasonia* [59], and the discovery of *osk* in this ant species strongly indicates that the absence of *osk* in the bee genome is a derived state.

We also analyzed the molecular basis of maternal germ plasm formation in the beetle *Acanthoscelides obtectus*, which, like *Nasonia*, but unlike *Tribolium*, produces an oosome and pole cells [32]. Like *Tribolium* and many other beetle species, *Acanthoscelides* possesses teletrophic ovarioles. In this type of oogenesis, a common pool of nurse cells is located at the anterior of the ovariole, which is connected with progressively maturing oocytes toward the posterior by actin and microtubule-rich structures called trophic cords [60]. In early oogenesis, Vas protein is highly enriched around the surface of the oocyte nucleues (Figure 8C). The presence of Vas protein is also detected in the nurse cells and trophic cords. In more mature oocytes, Vas protein is strongly enriched at the posterior pole, where the oosome will be formed (Figure 8D). This indicates that, despite employing a mode of oogenesis quite divergent from that seen in *Nasonia* and *Drosophila*, this beetle possesses similar capabilities for directing the localization and assembly of the germ plasm components to the posterior pole.

This is in contrast to *Tribolium*, where Vas protein is never found in a localized pattern in later oocytes (Figure 8F) despite its presence in the cytoplasm of early oocytes and in the trophic cords (Figure 8E), correlating well with the absence of pole cells and maternal germ plasm in this species. Based on the similarity of the pattern of Vasa protein accumulation in *Acanthoscelides* to the *osk* dependent Vas localization patterns in *Nasonia* and *Drosophila*, we predict that an *osk* ortholog is present in the genome of *Acanthoscelides*, and that it functions in recruiting Vas protein to the posterior pole of the oocyte and in assembling the oosome similar to its orthologs in *Nasonia* and *Drosophila*. Attempts to clone *osk* from the beetle by degenerate PCR have so far failed, and transcriptome or genome sequencing may be required to resolve this question.

## Discussion

### The origin of germ plasm and pole cells in holometabolous insects

**Figure 9:**
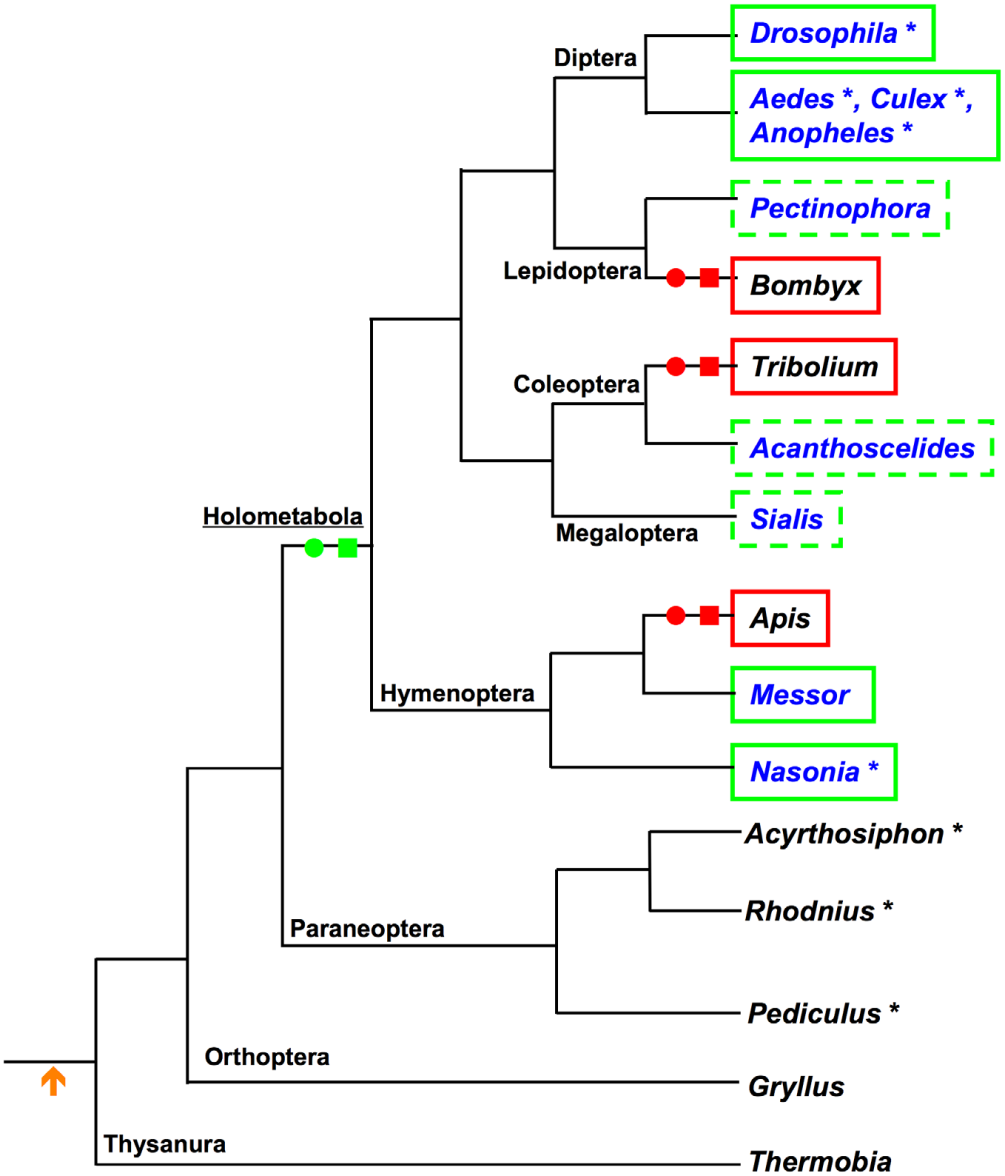
Phylogenetic pattern of losses and gains of maternal germ plasm, pole cells, and *oskar* among the insects. Genus names in blue are those in which maternal germ plasm and pole cells have been described. Asterisks indicate a sequenced genome. Green boxes indicate confirmed presence of *osk*. Red boxes indicate apparent absence of *osk* in the genome. Orange arrow indicates the ancestral use of zygotic induction of germline fate among insects. Green circles and squares indicate the proposed evolutionary origin of *osk* and maternally synthesized germ plasm, while red circles and squares indicate the proposed loss of these features, respectively. Tree was drawn based on the phylogenetic relationships described in [41], [59], [75].

Taken together, our results reveal a new picture for the origin and evolution of *oskar*, maternally provisioned germ plasm, and pole cells. We propose that the origins of these features represent evolutionary novelties of the Holometabola in relation to the rest of the insects, and that the appearance of the latter two features is strongly correlated with the presence of *osk* (Figure 9). Our conclusions are based on: (1) the presence of *osk* orthologs in the genomes of *Nasonia* and *Messor*, two distantly related hymenopteran species that also both have maternal germ plasm and pole cells; (2) the molecular and developmental similarity of the germ plasm of *Acanthoscelides* to that of *Drosophila* and *Nasonia*, which is consistent with the presence of an *osk* ortholog in this beetle; (3) the conserved interactions of Nv-Osk with upstream regulators (such as Nv-Bruno and Nv-Hrp48) and downstream partners (such as Nv-Vas and Nv-Tud), which indicate that a protein interaction network centered on Osk for generating maternal germ plasm and pole cells was present at the latest in the most recent common ancestor of the Hymenoptera and Diptera (which, based on current phylogenies would also be the common ancestor of all Holometabola) (Figure 9); and finally (4) the absence of maternal germ plasm, pole cells and *osk* in hemimetabolous insects, suggesting that the absence of these features is ancestral for the insects (Figure 9), and that these features likely arose after the divergence of the Holometabola from its sister group the Paraneoptera (true bugs, lice, and thrips).

The mapping of our findings on the insect phylogeny also indicates that *Apis*, *Tribolium*, and *Bombyx* may have lost these characters through independent evolutionary events (Figure 9). In addition, the correlation of the loss of maternal germ plasm and pole cells with the absence of *oskar* in these species (Figure 9), indicate that *osk* is a key factor in the evolution of germline determination mechanisms in the Holometabola.

Since production of the germline is a critical event in development and evolution, it is surprising that dramatic changes in how this cell fate is established have occurred several times in insect evolution. Such transitions could have been facilitated if redundant mechanisms for generating the germline existed in the ancestors of lineages that eventually lost the ability to maternally specify the germline.

In *Drosophila* there appears to be no remaining inductive capability: if pole cells are not produced, or are destroyed before reaching the gonad, the resulting fly is sterile. However, this is not the case in all insects. Destruction or removal of the oosome from the embryo of the wasp *Pimpla turionellae* resulted in the complete absence of pole cells, consistent with the role of the oosome in generating these cells. In spite of this, when embryos subject to these manipulations were examined later, a majority appeared to have germ cells populating the late embryonic gonads [61]. As *Pimpla* is a close relative of ants and bees, it is possible that both maternally provisioned germ plasm and the ability to zygotically induce germline fate coexisted in an ancestor of *Apis*, and the loss of the former capability thus may not have had dire consequences for the fecundity of species within the lineage leading to *Apis*. Once the presence of pole cells and maternal germ plasm was no longer selected for, it may have been relatively easy to lose *osk*, as long as another strategy for either localizing posterior *nanos*, or another mechanism for patterning the posterior is present.

The question of why an insect would lose the capacity to produce pole cells is also difficult to address directly. The likelihood that maternal provisioning of germline determinants evolved independently multiple times among animals [1], [2] implies that this strategy for germline determination has, at least under certain circumstances, selective benefits. Reciprocally, the multiple independent losses of this strategy indicate that, in other circumstances, zygotic induction may be favored. Broader sampling of germline specification strategies among the animals could shed light on the possible ecological or embryological traits correlated with the retention of or transition away from maternal synthesis of germline determinants and early segregation of the germ cell fate.

### The origin of *oskar*

Our finding that Oskar was a critical innovation for the transition to the maternal inheritance mode of germline determination in insects leads to the question of how such a novel protein could have been invented.

The strong similarity of the N-terminus of Nv-Osk to the N-terminus of Tdrd-7 orthologs found throughout the Metazoa, indicates that the origin of *osk* involved the duplication and divergence of this locus in an ancestor of the Holometabola. However, unlike *tdrd-7* genes, *osk* orthologs lack Tudor domains toward the C-terminus, and rather have a domain with structural similarity to SGNH/GDSL class hydrolases. Since such a domain is not found in Tdrd-7 orthologs, it may be that *osk* arose by a fusion of a *tdrd7* paralog, and a gene possessing a hydrolase domain.

While proteins of the SGNH/GDSL hydrolase family are found in all insect species, Osk orthologs show no significant homology to these sequences in BLAST analyses (E-value cutoff = 10). Rather, the highest scoring non-Oskar BLAST hits for the C-terminal portion (i.e., excluding the first 100 amino acids) of Osk proteins are often SGNH/GDSL hydrolases of Bacteria (e.g., Mp-Osk finds ZP_05979902.1 from *Subdoligranulum variabile* at an E-value of 0.17, and Cp-Osk finds YP_001491067.1 from *Arcobacter butzleri* at an E-value of 0.006). These observations raise the possibility that *osk* could have arisen by the combination of horizontal gene transfer from bacteria and gene fusion events. The fact that horizontal gene transfer from endosymbiotic bacteria occurs in insects is now well established [38], [62], and a source for a potential horizontal transfer could be the endosymbionts that are tightly associated with the early germ cells and gonads of many insect species (e.g., [63], [64]).

While the most parsimonious explanation for the observed distribution of *osk* orthologs among the Holometabola is that there was a single origin for this gene in a common ancestor of the holometbolan clade, we cannot formally exclude the possibility that the similarity in structure and function between the hymenopteran and dipteran Osk sequences was the result of two lineage specific events of convergent evolution responding to independent instances of selective pressure to establish cytoplasmic inheritance of germline components. However, it seems highly unlikely that the molecular events required to invent a novel gene such as *osk* would occur in almost identical ways twice in evolution before a different solution is found, let alone the unlikelihood of such a gene being fixed in a population, and then subsequently integrated into a novel regulatory network.

However, the invention of a novel factor required for cytoplasmic inheritance of germ plasm components may not be an occurrence unique to the Holometabola. In zebrafish, the *bucky ball* gene has an *osk*-like function in generating maternal germ plasm, but is molecularly unrelated to *osk*, and is only found in vertebrate genomes [11], [65]. This indicates that there is nothing intrinsic in the primary structure of Osk protein that is required for maternal assembly of germ plasm, and that there are many possible solutions to the problem of generating this substance. Further sampling of metazoan germline establishment strategies will give insight into how common the generation of novel genes is in the process of evolving maternally generated germ plasm.

### The origin of a protein regulatory network for restricting germ plasm production to the posterior pole

The process of maternal germ plasm assembly must be precisely controlled, and abnormalities in this process result in deep and sometimes spectacular consequences for the embryonic anterior-posterior axis [8]. Based on our results with *Nv-bruno* and *Nv-hrp48*, a common mechanism to spatially regulate *osk* localization and translation was likely already present at the origin of the Holometabola. This, along with the fact that factors such as Vas and Tud have conserved roles downstream of Osk in *Nasonia*, indicates that a complex protein interaction network for localized production of germ plasm during oogenenesis existed in a common ancestor of the Holometabola. This raises the question as to when during evolution has this network been assembled, and through which molecular mechanisms.

Proteins downstream of Osk, such as Tud, Vas, and Nos, have conserved roles in the specification and function of germ cells throughout the Metazoa, including those without maternal specification of the germline [11], and therefore are able to function without Osk to generate germ cell characteristics. Similarly, the proteins upstream of Osk, such as Bruno, Hrp48, and Staufen, are also highly conserved throughout the metazoa, and have conserved functions in mRNA localization and translational control in a variety of cellular contexts outside of the germline. Thus, Osk seems to have been intercalated between two ancient pre-existing regulatory networks. The position of Osk as the nexus between these two networks allows its specific and precisely controlled function in specifying the germline fate.

The fact that both the up- and downstream networks were already well established before the evolution of *osk* indicates that relatively few evolutionary steps may have been required to integrate Osk between them. In addition, since Osk is at least partially derived from a *tdrd7/5-*like gene, orthologs of which have well described functions in the germline in vertebrates and invertebrates, the ancestral Osk may have been predisposed to interact with other germ plasm components.

The localization of *osk* likely also had an evolutionary antecedent, as the presence of posteriorly localized patterning factors has been detected in some hemimetabolous species, e.g., [64], [66]. Since germ cells arise at the posterior pole just after gastrulation in some hemimetabolous species [16], [67], [68], it is possible that factors that predispose posterior nuclei to take germline fate are also localized at the posterior pole in these species. The molecular nature of any such factor, and whether its role is direct or indirect, remains to be determined. Testing the function and regulation of orthologs of genes both up- and downstream of *osk* in hemimetabolous, and other holometabolous, insect species should give insights into the functioning of the ancestral germline regulatory network, and could provide further clues as to how *osk* could have been integrated into it.

## Materials and Methods

A BLAST based strategy was used to identify potential *osk* orthologs in sequenced insect genomes (see ). The following databases were searched: for *Bombyx mori* , Silkworm Genome Assembly at silkdb.org [69]; for *Tribolium castaneum*, BeetleBase3_NCBI_DB at beetlebase.org [70]; for *Nasonia vitripennis*, Nasonia Scaffolds Assmebly Nvit_1.0 at hymenopteragenome.org/nasonia/ [71]; For *Apis melifera*, Scaffolds Assembly 2 at hymenopteragenome.org/beebase/; for *Acyrthosiphon pisum*, genome (reference only) at http://www.ncbi.nlm.nih.gov/projects/genome/seq/BlastGen/BlastGen.cgi?taxid=7029; for *Rhodnius prolixus*, *Harpegnathos saltator* and *Camponotus floridanus*, species-specific Whole-genome shotgun reads (wgs) databases were selected at blast.ncbi.nlm.nih.gov; for *Culex pipiens* Assembly CpipJ1- Johannesburg Strain, Supercontigs at http://cquinquefasciatus.vectorbase.org/Tools/BLAST/, and for *Pediculus humanus*, Assembly PhumU1, Supercontigs - USDA Strain at http://phumanus.vectorbase.org/Tools/BLAST/. These databases were queried using tblastn with default parameters (except the E-value cut off was raised to 10 where necessary) with the following Oskar protein sequences: NP_731295.1 (*Drosophila*), XP_001848641.1 (*Culex*), ADK94458.1 (*Nasonia*), and HM992570 (*Messor*). To identify *osk* orthologs among EST sequences, the same query sequences and tblastn parameters were used at blast.ncbi.nlm.nih.gov to search the (est others) database.

Templates for probe and dsRNA production were generated as in [72]. dsRNA was produced using T7 Megascript kit (Ambion) following manufacturers instructions. Fragments used to generate dsRNA and probes were as follows: *Nv-osk—*bases 161–843 of Genbank accession HM535628.1, *Nv-bruno—*bases 534–1394 of Genbank accession XM_001605096.1, *Nv-vasa—*bases 827–1613 of Genbank accession XM_001603906.1, *Nv-hrp48—*bases 434–1727 of Genbank accession XM_001600216.1, *Nv-tudor—*bases 6053–6811 of Gnomon model hmm120984.

RNAi experiments were performed as described in [39]. dsRNAs were used at the following concentrations: *Nv-osk*- 3.5 mg/mL, *Nv-vasa*- 3 mg/mL, *Nv-bruno*-2.5 mg/mL, *Nv-hrp48*- 1.0 mg/mL, *Nv-tudor*- 2.5 mg/mL. Knockdown was confirmed by comparing expression levels of the gene of interest in ovaries of wild-type wasps to those from pRNAi treated wasps. All genes showed clearly reduced levels of expression after their corresponding dsRNA injections, but the degree of knockdown was variable from egg chamber to egg chamber.

RACE PCR for *Nv-osk* was performed using the SMART-RACE kit (Takara) according to manufacturer's instructions.

The *Nasonia* Vasa antibody was generated using the custom peptide antibody service of Sigma-Genosys with the peptide CVLRHDTMKPPGERQ as the antigen. It was used at 1∶500, and detected using anti-rabbit Alexa 555 (Invitrogen) at 1∶750

The cross reactive *Drosophila* Vasa antiserum used in *Tribolium* and *Acanthoscelides* was a generous gift from Akira Nakamura [73]. It was used at 1∶1000 and detected as above.

*in situ* hybridization and immunohistochemistry were performed as described in [51].

The ant *osk* sequence was found in the course of a genome sequencing project (unpublished) and cloned from the ant *Messor pergandei* using the following primers: AntOsk forward ATGGAWGAAACAGTGGCATTRRTMAAAT and AntOsk reverse GGAACCARTCGTAWTCYGTRRTRTACGTT. The cloned 1057 base pair fragment was validated by sequencing, submitted to Genbank with accession # HM992570 and used to generate an antisense Digoxigenin labeled probe for *in situ* hybridization. Embryos and ovaries of *Nasonia* and the beetles were collected and fixed as in [72]. Ant embryos and ovaries were prepared and stained for *osk* mRNA as described in [74].

## Supporting Information

Figure S1 Expression of components of the maternal germ plasm regulatory network in *Nasonia* ovarioles. A: *Nasonia vasa* expression. B: *Nasonia tudor* expression. C: *Nasonia bruno* expression. D: *Nasonia hrp48* expression. Arrows in A indicate the higher levels of expression in the most anterior nurse cells. Scale bar represents 0.1 mm. All ovarioles are oriented with anterior up.
(TIF)

Table S1 Identification of Oskar orthologs in insect genomes. Potential *osk* orthologs were searched for in the genomes of insects using BLAST . Details of the sequences and databases used and the parameters employed can be found in the Materials and Methods section. Red boxes indicate a hit against a putative *osk* ortholog, blue boxes indicate hits against non-Oskar tejas/lotus domain containing genes. N/A indicates that no hits were obtained using and E-value cutoff of 10. Only hits with E-values less than one are shown, except where the best hit in the searched genome for a particular Osk ortholog is greater than one. The values in the first row of each genome searched are the E-values of the best hit, and any other hit with an E-value less than 1, returned by the corresponding Osk ortholog. In the second row of each searched genome field, the Genbank or genome database accession number of either a predicted gene corresponding to the genomic hit, or, if no gene is predicted, the genomic coordinates are shown. The best, and significant hits were then used as queries against the *Drosophila* genome, and the resulting CG identifiers are shown in the third row under each genome searched, and the E-values of the matches are shown on the fourth row. Since the *Nasonia* and *Messor* Osk sequences can detect the rapidly diverging *osk* sequence of *D. melangaster*, we would expect that these sequences should be able to find *osk* sequences in the genomes of *Apis*, *Bombyx*, and *Tribolium*, were they present, unless the evolution at the *osk* loci species were independently accelerated in each of their lineages beyond the rate seen in the fly. Due to the nature of whole genome shotgun sequencing, we cannot exclude that genomic regions including *osk* orthologs were coincidentally missed in the genomes where no *osk* is found. * In these cases the *Culex* sequences did not give significant results, and the results shown are from using the Osk ortholog from the closely related mosquito species *Aedes aegypti*. Using the *Aedes* sequence in other genomes did not give significantly different results. ** The genomic region surrounding the region showing homology to Osk was used as input into FgenesH using the *Apis* model at http://linux1.softberry.com/ to predict an Osk sequence, that was then used as a query against the fly genome. *** The genomic region surrounding the region showing homology to Osk was used as input into FgenesH+ using the *Apis* model and Nv-Osk protein sequence at http://linux1.softberry.com/ to predict an Osk sequence, which was then used as a query against the fly genome.
(XLS)
