# Supplementary figures and images for: The Phylogenetic Origin of oskar Coincided with the Origin of Maternally Provisioned Germ Plasm and Pole Cells at the Base of the Holometabola
Source: PLoS Genet. 2011 Apr 28;7(4):e1002029. doi: 10.1371/journal.pgen.1002029 (PMC3084197; doi:10.1371/journal.pgen.1002029)

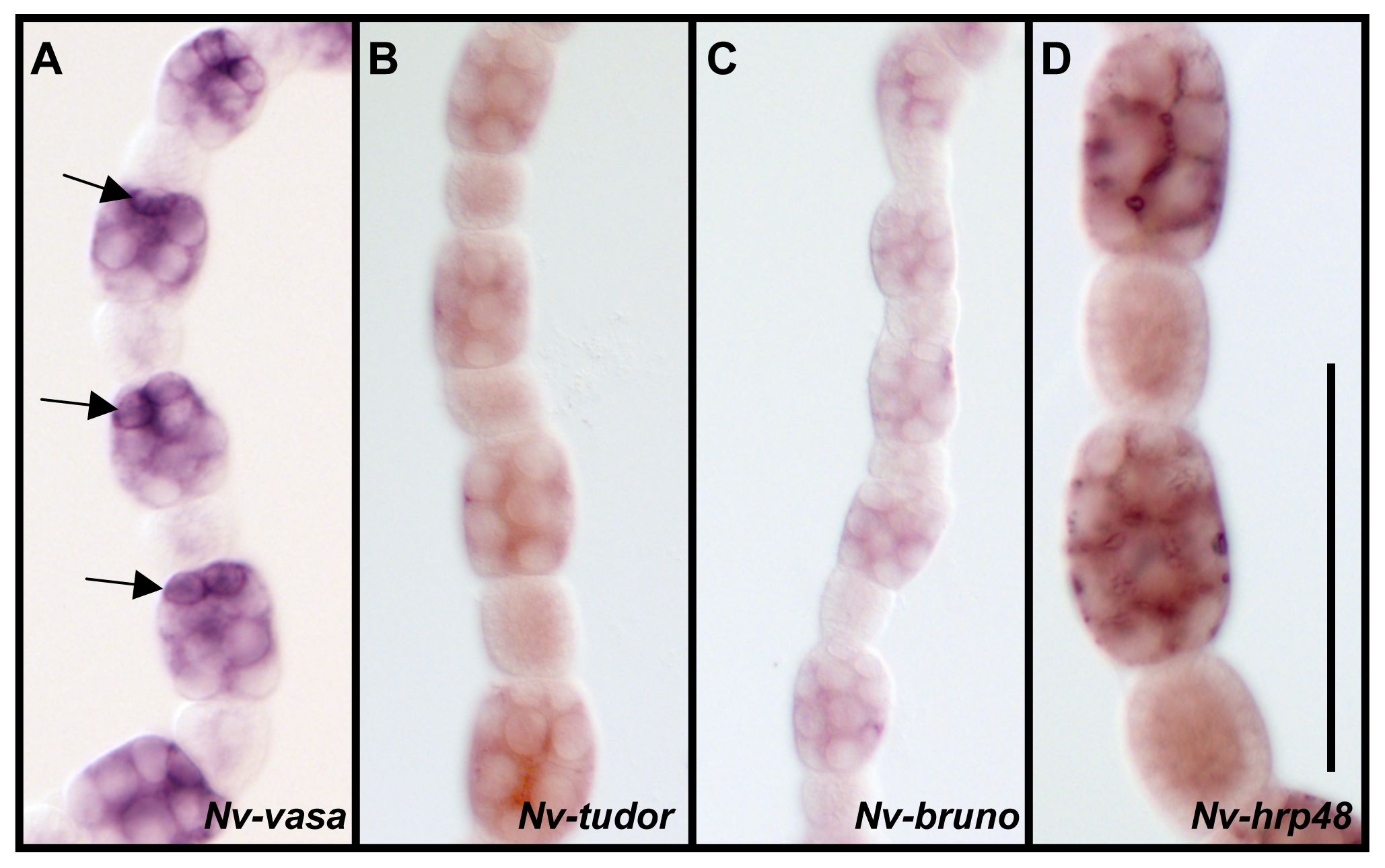

Supplement: Figure S1 — Expression of components of the maternal germ plasm regulatory network in Nasonia ovarioles. A: Nasonia vasa expression. B: Nasonia tudor expression. C: Nasonia bruno expression. D: Nasonia hrp48 expression. Arrows in A indicate the higher levels of expression in the most anterior nurse cells. Scale bar represents 0.1 mm. All ovarioles are oriented with anterior up. (TIF) [file pgen.1002029.s001.tif]
